# Myotonic Dystrophy Initially Presenting as Tachycardiomyopathy Successful Catheter Ablation of Atrial Flutter

**DOI:** 10.4061/2010/383852

**Published:** 2010-08-24

**Authors:** S. Asbach, K. J. Gutleben, P. Dahlem, J. Brachmann, G. Nölker

**Affiliations:** ^1^II. Medizinische Klinik, Klinikum Coburg, 96450 Coburg, Germany; ^2^Medizinische Universitätsklinik, Innere Medizin III, Abt. für Kardiologie und Angiologie, 79106 Freiburg, Germany; ^3^Klinik für Kinder und Jugendliche, Klinikum Coburg, 96450 Coburg, Germany; ^4^Herz- und Diabeteszentrum Nordrhein-Westfalen, 11 Georgstraße, 32545 Bad Oeynhausen, Germany

## Abstract

Myotonic dystrophy is a genetic muscular disease that is frequently associated with cardiac arrhythmias. Bradyarrhythmias, such as sinus bradycardia and atrioventricular block, are more common than tachyarrhythmias. Rarely, previously undiagnosed patients with myotonic dystrophy initially present with a tachyarrhythmia. We describe the case of a 14-year-old boy, who was admitted to the hospital with clinical signs and symptoms of decompensated heart failure and severely reduced left ventricular function. Electrocardiography showed common-type atrial flutter with 2 : 1 conduction resulting in a heart rate of 160 bpm. Initiation of medical therapy for heart failure as well as electrical cardioversion led to a marked clinical improvement. Catheter ablation of atrial flutter was performed to prevent future cardiac decompensations and to prevent development of tachymyopathy. Left ventricular function normalized during followup. Genetic analysis confirmed the clinical suspicion of myotonic dystrophy as known in other family members in this case.

## 1. Introduction

Pediatric patients suffering from myotonic dystrophy frequently exhibit electrocardiographic abnormalities, most often sinus bradycardia and conduction disturbances [1, 2]. These diagnoses are usually established once the diagnosis of myotonic dystrophy has been made. Tachyarrhythmias are infrequently found not only in pediatric patients with myotonic dystrophy, but generally in children. These are usually supraventricular arrhythmias and may be divided into those based on congenitally existing, primary arrhythmogenic substrates, such as accessory pathways, and those due to acquired, secondary arrhythmogenic substrates related to congenital heart disease [3]. Patients in the pediatric population who present with atrial flutter usually suffer from underlying heart disease or have already undergone surgery for a congenital heart defect [3]. Only in rare cases, the workup of these patients does not reveal an underlying structural heart disease and thus is more challenging. 

## 2. Case Report

**Figure 1:**
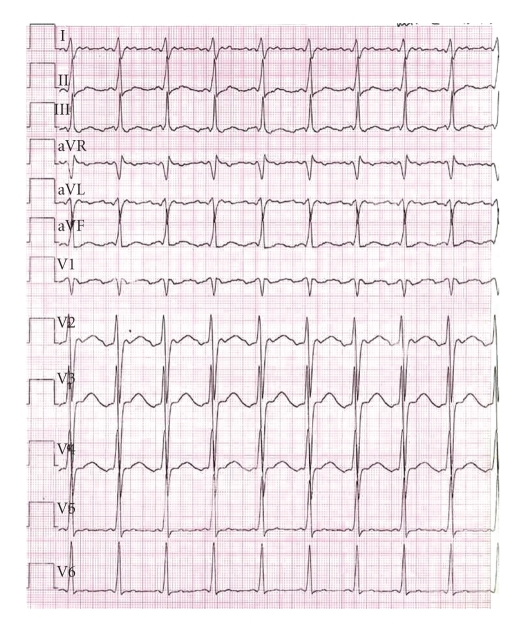
The initial ECG (50 mm/s) shows supraventricular tachycardia with 2 : 1 conduction and a resulting heart rate of 160 bpm. Negative deflections of the P-waves in leads II, III, and aVF suggest the presence of counter-clockwise, isthmus-dependent atrial flutter.

A 14-year-old boy was taken to the outpatient pediatric department after feeling sick following climbing, with vomiting and presyncope. He also complained of recurrent palpitations within the previous two weeks. The initial electrocardiogram showed common type atrial flutter with 2 : 1 conduction resulting in a heart rate of 160 bpm (Figure 1). The previous medical history was unremarkable. Father and sister of the patient were known to suffer from myotonic dystrophy, but according to the patient's parents, repeated neurologic examinations in the past did not show signs of the disease.

Upon admission to the hospital, echocardiography showed severely reduced left ventricular function with fractional shortening of 14%, but no signs of a congenital heart defect. Clinical examination showed a facies myotonica. Initiation of beta-blocking agents (metoprolol succinate 23.75 mg) was unable to slow the heart rate sufficiently, so that electrical cardioversion was performed resulting in sinus tachycardia with 120 bpm. Further recompensation was achieved by adding diuretics (repetitive i.v. injections of 10 mg furosemide) and angiotensin converting enzyme inhibitors (captopril 3 × 6.25 mg). The PQ interval after cardioversion was within normal range (166 ms).

**Figure 2:**
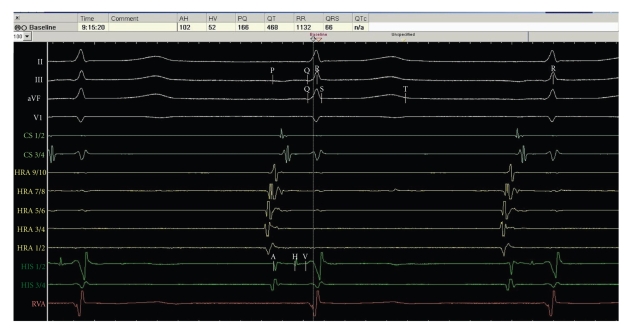
Intracardiac electrograms showing normal conduction times during sinus rhythm, especially without infrahisian delay. PQ:166 ms, QRS: 66 ms, AH: interval 102 ms, HV-interval: 52 ms, HRA: high right atrium, HIS: His-bundle electrogram, RVA: right ventricular apex, and CS: coronary sinus electrogram.

Subsequently, catheter ablation of the arrhythmia was performed. A bidirectional block of the cavo-tricuspid isthmus was achieved without complications. Infra-Hisian conduction time was within normal range (52 ms, Figure 2). Ventricular arrhythmias could not be induced with pacing from the right ventricular apex or the right ventricular outflow tract with up to three extra stimuli. No atrial arrhythmias could be induced by programmed stimulation in high right atrium.

Because of the family history, as well as an elevated creatin-kinase (1832 U/l), genetic analysis was performed which confirmed the suspicion of myotonic dystrophy of Curschmann-Steinert type 1. During followup, left ventricular function continuously improved (fractional shortening 29%) and no recurrence of the arrhythmia could be observed. Magnetic resonance imaging of the heart failed to show signs of pericarditis, myocarditis, or cardiac involvement of myotonic dystrophy, such as myocardial thickening or isolated left ventricular abnormal trabeculation [4].

## 3. Discussion

Arrhythmias, especially in the pediatric population, should prompt for a search for the underlying disease. In most cases, either a congenitally existing primary arrhythmogenic substrate (such as an accessory pathway) or sequelae of congenital heart disease, or its surgical correction can be identified as the cause. Interventional electrophysiological treatment can be performed in these patients with high success rates [3, 5]. The workup in cases such as the ones presented with a primary rhythm disorder without an obvious cardiac defect is more challenging. 

Cardiac involvement is an integral part of Curschmann-Steinert's myotonic dystrophy, the most reported manifestations having various conduction defects [1, 2, 6]. A recent study reported cardiac involvement in 77.4% of cases, 64% having first-degree atrioventricular block and 32% bundle branch block [7]. The severity of cardiac involvement in this study was not linked to severity of the neurological abnormalities. Pacemaker implantation is recommended to these patients as secondary prevention of sudden death, but may also be appropriate to patients with documented infrahisian conduction abnormality as defined as an H-V interval ≥70 ms [8]. When present, the most constant findings in cardiac histopathology are myocyte hypertrophy, interstitial fibrosis, and fatty infiltration [9]. These may serve as the basis for unidirectional block within the cardiac tissue, which is the prerequisite for re-entry, which again is necessary for initiation of atrial flutter.

While usually the neurological manifestations lead to the diagnosis of myotonic dystrophy, there are case reports of patients who initially present with symptoms of cardiac involvement [10]. 

It is noteworthy that in our patient no signs of atrioventricular conduction delay were present in surface ECG, and the electrophysiology study showed normal conduction times.

Common-type atrial flutter is a well-defined arrhythmia with a critical zone of slow conduction at the right atrial isthmus between the inferior vena cava and the inferior aspect of the tricuspid annulus. Catheter ablation is the therapy of choice in the adult population, and high success rates of this arrhythmia were also reported in the pediatric population [3, 5]. 

The clinical course in the presented case with the improvement of cardiac function after rhythm control and the structurally normal heart on echocardiography and magnetic resonance tomography make it very likely that the rhythm disorder itself led to the clinical deterioration. It can be speculated that undetectable structural cardiac abnormalities linked to the underlying myotonic dystrophy (as described above) are the substrate for the rhythm disorder. Curative catheter ablation is an appropriate means of treatment and helps prevent future cardiac decompensations and the development of tachymyopathy [11]. 

Cardiac arrhythmias in children without detectable structural cardiac abnormalities should be investigated for the presence of myopathy. Close cooperation of pediatric neurologists, pediatric cardiologists, and clinical electrophysiologists is necessary to offer best medical care.
